# Machine Learning based Radiomics from Multi-parametric Magnetic Resonance Imaging for Predicting Lymph Node Metastasis in Cervical Cancer

**DOI:** 10.2174/0115734056376718250904221020

**Published:** 2025-09-18

**Authors:** Jing Liu, Mingxuan Zhu, Li Li, Lele Zang, Lan Luo, Fei Zhu, Huiqi Zhang, Qin Xu

**Affiliations:** 1 Departments of Gynecology, Clinical Oncology School of Fujian Medical University, Fujian Cancer Hospital (Fujian Branch of Fudan University Shanghai Cancer Center), Fuzhou 350014, Fujian, China

**Keywords:** Cervical cancer, Lymph node metastasis, Machine learning, Magnetic resonance imaging, Kaplan-meier, Suitable treatment

## Abstract

**Introduction:**

Construct and compare multiple machine learning models to predict lymph node (LN) metastasis in cervical cancer, utilizing radiomic features extracted from preoperative multi-parametric magnetic resonance imaging (MRI).

**Methods:**

This study retrospectively enrolled 407 patients with cervical cancer who were randomly divided into a training cohort (n=284) and a validation cohort (n=123). A total of 4065 radiomic features were extracted from the tumor regions of interest on contrast-enhanced T1-weighted imaging, T2-weighted imaging, and diffusion-weighted imaging for each patient. The Mann-Whitney U test, Spearman correlation analysis, and selection operator Cox regression analysis were employed for radiomic feature selection. The relationship between MRI radiomic features and LN status was analyzed using five machine-learning algorithms. Model performance was evaluated by measuring the area under the receiver-operating characteristic curve (AUC) and accuracy (ACC). Moreover, Kaplan–Meier analysis was used to validate the prognostic value of selected clinical and radiomic characteristics.

**Results:**

LN metastasis was pathologically detected in 24.3% (99/407) of patients. Following a three-step feature selection, 18 radiomic features were employed for model construction. The XGBoost model exhibited superior performance compared to other models, achieving an AUC, accuracy, sensitivity, specificity, and F1 score of 0.9268, 0.8969, 0.7419, 0.9891, and 0.8364, respectively, on the validation set. Additionally, Kaplan−Meier curves indicated a significant correlation between radiomic scores and progression-free survival in cervical cancer patients (*p* < 0.05).

**Discussion:**

Among the machine learning models, XGBoost demonstrated the best predictive ability for LN metastasis and showed prognostic value through its radiomic score, highlighting its clinical potential.

**Conclusion:**

Machine learning-based multi-parametric MRI radiomic analysis demonstrated promising performance in the preoperative prediction of LN metastasis and clinical prognosis in cervical cancer.

## 1 INTRODUCTION

Cervical cancer ranks as the fourth most frequently diagnosed cancer and the fourth leading cause of cancer-related deaths among women [1, 2]. Preoperative clarification of lymph node (LN) status in cervical cancer is crucial, as it facilitates treatment planning and prognosis assessment [3-5]. Several pathological factors have been identified as being correlated with lymph node metastasis (LNM) in cervical cancer, including the expression of programmed death ligand 1 (PD-L1) and TIMELESS [6, 7]. However, these pathological markers can only be assessed postoperatively, limiting their utility in preoperative decision-making. Therefore, there is a crucial need for noninvasive and precise LNM prediction to ensure accurate patient staging in cervical cancer and to subsequently guide the selection of the most suitable treatment.

In clinical practice, diverse imaging methods such as ultrasound (US), computed tomography (CT), and magnetic resonance imaging (MRI) are used to identify LN status in cancer patients [8]. MRI provides more intricate anatomical details for diagnosis and encompasses a richer array of texture information in its images than US and CT [9]. Despite these advantages, imaging methods face constraints in evaluating LNM in cervical cancer and are not recommended as the primary basis for determination, due to limited sensitivity and accuracy [10]. Radiomics enables the extraction and quantification of features from medical images to assess pixel-level characteristics that are imperceptible to the human eye [11]. Existing studies have also demonstrated a correlation between radiomic features within the primary tumor region and LNM [12-15].

Nowadays, the application of the Internet of Things (IoT) in medicine has become increasingly widespread, and its integration with artificial intelligence (AI) further enhances healthcare efficiency and diagnostic accuracy [16]. AI and its subfields, particularly machine learning (ML) and deep learning (DL), are increasingly expanding their applications in the medical field. These technologies have demonstrated significant potential in various domains, including disease diagnosis, treatment planning, and prognosis prediction [17]. By extracting and analyzing radiomic features from medical images and integrating them with ML algorithms, multiple predictive models have been developed, offering novel auxiliary tools for clinical diagnosis and treatment [18]. According to previous studies [19-24], radiomics and ML can be used for subtype classification, survival prediction, and for assessing the impact of different diseases. Predicting LNM using MRI features in cervical cancer poses a contemporary challenge. Several radiomics studies have been conducted to address this problem [25, 26]. Wang *et al.* [25] constructed an ML model with a Support Vector Machine (SVM) based on T2-weighted imaging (T2WI) and diffusion-weighted imaging (DWI), which predicted LNM in early-stage cervical cancer. Nevertheless, research on integrating radiomics and ML for LNM prediction and prognosis assessment in cervical cancer remains limited.

This study aims to investigate the diagnostic performance of machine-learning models based on multi-parametric 3D radiomics for preoperative cervical cancer LNM and to evaluate the role of radiomic features in predicting cervical cancer prognosis.

## 2 MATERIAL AND METHODS

### 2.1 Patients

This study was approved by the medical ethics committee review board of the Fujian Cancer Hospital (K2024-210-01), and no informed consent requirements were necessary. Clinical and multi-parametric MRI data were collected from patients with cervical cancer confirmed by pathology at Fujian Cancer Hospital from February 2009 to June 2013. The primary outcome of this study was LNM, determined by pathological results or radiological evidence of nodal progression during follow-up. The secondary outcome was overall survival (OS), defined as the time from diagnosis to death from any cause or last follow-up. Patients were enrolled based on the following inclusion criteria: (1) 18 years or older; (2) performance status, Eastern Cooperative Oncology Group (ECOG) ≤2; (3) histolo gically confirmed squamous carcinoma, adenocarcinoma, or adenosquamous carcinoma of the cervix; (4) International Federation of Gynecology and Obstetrics (FIGO) stage (2018) from IB3 to IIB. The exclusion criteria were: (1) incomplete information or severe imaging artifacts in the MRI images; (2) lesion diameter <5 mm on MRI images. Ultimately, 407 patients with cervical cancer were enrolled in the study. According to the proportion of 7: 3, all cervical cancer patients were divided into the training cohort and the validation cohort. The collected clinical data included age, macroscopic type, tumor size, postoperative pathology, degree of differentiation, depth of tumor invasion, corpus invasion, parametrial invasion, vaginal invasion, and LNM.

### 2.2 MRI Image Acquisition and Segmentation

Magnetic resonance (MR) images were obtained using a 1.5 Tesla MR system (SIGNA INFINITY TWINSPEED 1.5T, GE, Fairfield City, USA). Conventional MR scanning utilized an 8-channel phased-array surface coil pairs, while DWI scanning incorporated an external body surface coil. MRI sequences included axial T1-weighted fast spin echo (FSE; TR = 620 ms, echo train length = 3, FOV = 340 × 340 mm^2^), oblique sagittal T2-weighted fast recovery fast spin echo (FRFSE; TR = 3660 ms, TE = 86.6 ms, echo train length = 25, FOV = 300 × 300 mm^2^), oblique coronal T2-weighted FRFSE (TR = 3120 ms, TE = 92.3 ms, echo train length = 21, FOV = 320 × 320 mm^2^), axial diffusion-weighted imaging (DWI; TR = 5120 ms, TE = 55.2 ms, echo train length = 1, FOV = 340 × 340 mm^2^), sagittal contrast-enhanced 3D spoiled gradient echo (SGRE, LAVA; TR = 4.2 ms, TE = 2.0 ms, FOV = 300 × 300 mm^2^), and axial breath-hold contrast-enhanced 3D SGRE (LAVA; TR = 4.7 ms, TE = 2.2 ms, FOV = 380 × 380 mm^2^). DWI was performed before the intravenous injection of gadolinium. An intravenous injection of 0.2 mmol/kg body weight gadopentetate dimeglumine (Magnevist, Schering AG, Germany) was administered for the post-gadolinium series. Patients maintained a stable supine position during scanning to minimize motion artifacts and preserve image quality. The slice thickness and slice interval were both 4 mm. Raw image data were transferred to a postprocessing workstation for reconstruction and preprocessing, including denoising, correction, and normalization to ensure accurate radiomic analysis. Image standardization involved cropping the gross tumor volume (GTV), resizing all images to 32 × 256 × 256 based on GTV position, and resampling voxels to 1 × 1 × 1 mm^3^ using linear interpolation.

The region of interest (ROI) delineation was performed by two experienced professionals. One experienced radiologist (J.L, with 10 years of pelvic MRI reading expertise) manually outlined 3D tumor contours on axial slices using 3D Slicer software (version 4.11, https://www.slicer.org). The segmentation results were confirmed by another gynecologic oncology expert (Q.X, with 20 years of clinical experience). Both radiologists remained blinded to the clinical and histopathological data throughout this process. Finally, images and image masks were exported as 3D files in the Neuroimaging Informatics Technology Initiative (NIfTI) format.

### 2.3 Features Extraction and Selection

The radiomics feature extraction was performed in Python (version 3.8.3) using the Pyradiomics package (version 3.0.1, https://github.com/Radiomics/pyradiomics) [27]. Quantitative radiomic features were extracted from the tumor ROI in T1-weighted imaging (T1WI), T2WI, and DWI, including shape-based, first-order statistical, texture, and wavelet features.

Before further analysis, all the extracted radiomics features underwent standardization into a normal distribution with z-scores, effectively mitigating the differences in the value scales of the data [28]. In pursuit of selecting highly relevant and non-redundant features, a three-step method was applied. Firstly, the Mann–Whitney U test was conducted to select the features, retaining those with p<0.05 as significantly different. Secondly, we sequentially used Spearman correlation analysis to eliminate redundant radiomic features, with features exhibiting a Spearman correlation coefficient exceeding 0.9 being excluded. Finally, the Least Absolute Shrinkage and Selection Operator (LASSO) regression algorithm was utilized for further dimensionality reduction and optimized feature selection.

### 2.4 Development and Validation of ML–based Models

Based on the selected optimal feature subset, models were constructed using ML algorithms such as logistic regression (LR), Naïve Bayes (NB), support vector machine (SVM), adaptive boosting (AdaBoost), and extreme gradient boosting (XGBoost). LR learns a probabilistic model for binary classification by optimizing its parameters to align its predictions with the actual class labels as closely as possible. NB is a classification algorithm based on Bayes' theorem. The objective of SVM is to identify an optimal hyperplane that separates samples of different classes while maximizing the margin between the two classes. AdaBoost is an ensemble learning algorithm that constructs a strong classifier by combining multiple weak classifiers. XGBoost is based on the framework of boosting trees, constructing multiple weak learners sequentially, with each learner correcting the errors of its predecessor, thereby continuously optimizing predictive performance. The performance of each ML-based model was evaluated using the receiver operating characteristic curve (ROC), decision curve analysis (DCA), and calibration curves. The performance of the algorithms was also assessed in terms of accuracy, sensitivity, and specificity. Fig. (**1**) illustrates the study design and workflow.

### 2.5 Statistical Analysis

Feature extraction, feature selection, and training and validation of ML models were conducted using Python (version 3.6, https://www.python.org). To compare clinical characteristics between the training and validation groups, the t-test was applied for continuous variables, and the chi-square test (for groups with n=5) or Yates' corrected chi-square test (for groups with n=5) was employed for categorical variables. A p-value greater than 0.05 indicated no significant difference between the groups [29, 30]. The reliability of the radiomics scoring risk stratification system was evaluated through Kaplan-Meier curves. All results with a p-value less than 0.05 were considered statistically significant.

## 3 RESULTS

### 3.1 Patient Characteristics

Table **1** outlines the clinical and demographic characteristics of the training cohort (n=284) and the validation cohort (n=123). The median age of patients in both the training and independent validation cohort was 47±8 years. The number of patients with LNM was 68 (23.9%) in the training cohort and 31 (25.2%) in the validation cohort. The median follow-up time for surviving patients was 103 months for the training cohort and 110 months for the validation cohort. The results indicate no significant statistical differences in clinical characteristics between the two cohorts.

### 3.2 Feature Selection

A total of 4650 radiomic features were initially extracted from the ROIs of contrast-enhanced T1-weighted imaging (ceT1WI), T2WI, and DWI. Features highly correlated with LNM were selected using the Mann-Whitney U test (p<0.05), reducing the features to 1356. Subsequent Spearman correlation testing retained 92 features with coefficients exceeding 0.9. The correlation of variables was assessed using a heatmap (Fig. **S1**). Finally, LASSO regression identified 18 radiomic features most correlated with lymph node metastasis, and Table **2** details these features along with their LASSO coefficients.

### 3.3 Performance of Prediction Models

**Fig. (2):**
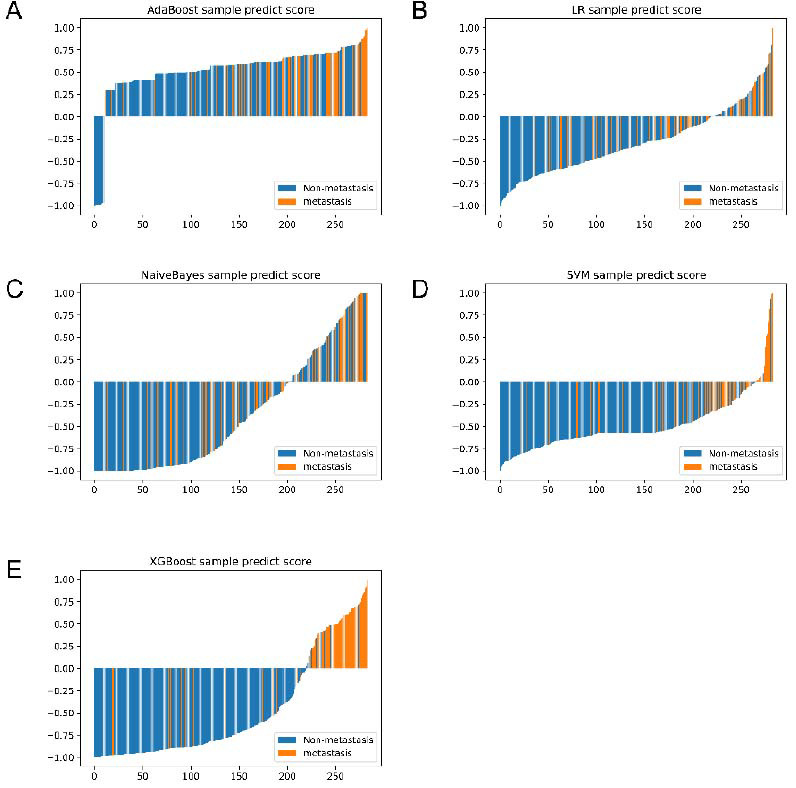
Comparisons of prediction scores for each patient when predicting the LNM status in the training cohort. AdaBoost, adaptive boosting (**A**); LR, Logistic Regression (**B**); NB, Naïve Bayes (**C**); SVM, support vector machine (**D**); and XGBoost, extreme gradient boosting (**E**). Red bars represent patients who developed LNM. Blue bars represent that LNM was negative.

Table **3** summarizes the predictive performance of all ML models. Notably, XGBoost exhibited superior performance, achieving a classification accuracy of 0.927 and an AUC of 0.897 for predicting the lymph node status of cervical cancer patients in the validation set. Its sensitivity, specificity, positive predictive value, negative predictive value, precision, and F1 score were 0.741, 0.989, 0.958, 0.919, 0.958, and 0.836, respectively. In comparison, other models showed varying levels of accuracy and AUC in the validation set: LR (0.667, 0.715), NB (0.724, 0.734), SVM (0.740, 0.791), and AdaBoost (0.683, 0.737). These findings underscore the superior predictive performance of the XGBoost model in cervical cancer lymph node status prediction. (Fig. **2A**-**E**) visually depicts the prediction scores for each patient's lymph node status, highlighting XGBoost's effectiveness in distinguishing between LN-negative and LN-positive groups.

**Fig. (3):**
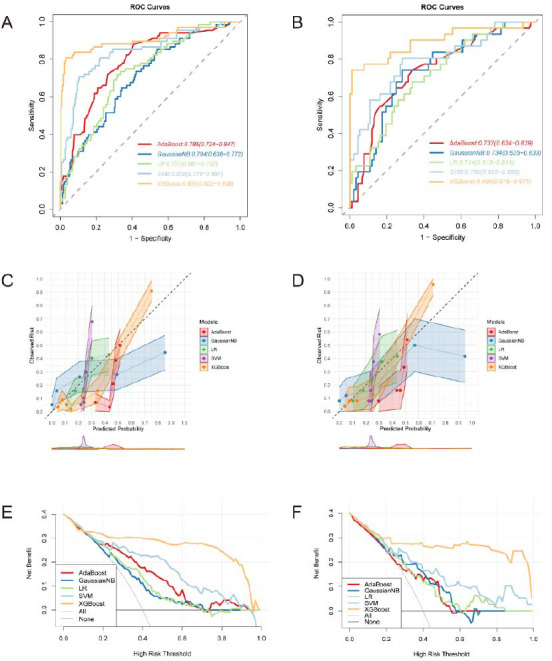
Receiver operating characteristic (ROC) curves, Calibration curves, and decision curves of machine-learning models. The ROC curves in the training cohort (**A**) and the validation cohort (**B**); calibration curves in the training cohort (**C**) and the validation cohort (**D**); decision curve in the training cohort (**E**) and the validation cohort (**F**).

For a clearer comparison of different models in predicting the lymph node status of cervical cancer, (Fig. **3A**, **B**) illustrate the AUCs for all models in both the training and validation cohorts. The DCA curve was used to evaluate the clinical value of these models (Fig. **3C**, **D**). Assuming no patients have LNM in cervical cancer, the solid black line (negative line) indicates that the net benefit is zero when no patient receives therapy. Conversely, the solid grey line (positive line) represents the net benefit when all patients with LNM receive therapy. The XGBoost model demonstrated higher net benefit compared to the two extreme lines (negative line and positive line) in both cohorts. Notably, XGBoost demonstrated significantly superior performance compared to the others across most threshold points. Calibration curves reflect the degree of consistency between the observed risk and predicted probabilities of a model. (Fig. **3E**, **F**) confirm that the XGBoost model exhibits the optimal level of consistency.

### 3.4 Radiomics Score and Prognostic Stratification

Furthermore, a radiomics score (RS) for each patient was calculated using the XGBoost model, allowing stratification into low-RS and high-RS subgroups. Kaplan-Meier analysis further validated the prognostic significance of the selected radiomic features. As shown in Fig. (**4**), the Kaplan-Meier curves revealed a significant difference in survival probabilities between the two risk subgroups (*p* = 0.005). Patients with a higher RS exhibited lower overall survival rates (HR = 1.9, 95% CI: 1.2–3.0, *p* = 0.006). Across the entire cohort, RS-based stratification was significantly associated with progression-free survival (PFS).

## 4 DISCUSSION

Cervical cancer is a significant health issue globally, with LNM being a critical factor affecting prognosis and treatment decisions [4]. Preoperative prediction of LNM in cervical cancer can guide clinical management, as current imaging techniques, such as CT and ultrasound often fall short in accuracy. Radiomics, by extracting detailed features from medical images, has emerged as a promising approach to improve diagnostic precision [31].

In this study, we developed and validated various radiomics-based ML diagnostic models for predicting LNM in cervical cancer using multi-parametric MRI. Leveraging preoperative MRI with multiple parameters, we extracted 4065 imaging features, ultimately selecting 18 features and constructing multiple ML diagnostic models. The XGBoost model demonstrated superior performance in predicting lymph node metastasis in the validation set, with an accuracy of 0.9268 and an AUC of 0.8969, outperforming the other models.

Radiomics analysis has been widely applied to improve tumor diagnosis, staging, and prognostic evaluation by extracting high-dimensional quantitative features from medical images [32-34]. Fang *et al.* [34] demonstrated that radiomics scores derived from MRI serve as prognostic biomarkers for early-stage cervical cancer patients. Kan *et al.* [35] utilized T2WI and ceT1WI to extract radiomic features and employed an SVM model(AUC(95% CI), 0.754(0584–0.924)) to predict LNM in early-stage cervical cancer. However, some studies have been limited by the use of features extracted from a single MRI sequence [36, 37] or have performed radiomics analysis on only 2D slices [38], potentially missing critical spatial information. In contrast, our approach involved using three common 3D MRI sequences in cervical cancer patients for feature extraction, allowing for the extraction of rich radiomic features and enhancing the model's reliability. Moreover, our systematic comparison of multiple ML models identified XGBoost as the most effective model, demonstrating strong and consistent performance across training and validation cohorts (AUC = 0.897 in the validation set), reinforcing the robustness of our methodology.

Our research utilized a diverse set of ML algorithms, including LR, NB, SVM, AdaBoost, and XGBoost. XGBoost is a gradient boosting decision tree algorithm known for its ability to handle high-dimensional datasets efficiently. It employs iterative decision tree training, L1/L2 regularization, and parallel processing, making it particularly suitable for complex classification tasks. Compared to SVM, which is effective for linearly separable data but may struggle with high-dimensional feature spaces, XGBoost demonstrated superior predictive accuracy (AUC = 0.897 vs. 0.791 for SVM). Similar findings have been reported in other oncological radiomics studies; for instance, Sheng *et al.* [23] demonstrated that an XGBoost-based model outperformed conventional ML models in predicting invasive ductal breast cancer subtypes, particularly in distinguishing triple-negative and non-triple-negative cases (AUC = 0.903). Song *et al.* [39] also used the XGBoost algorithm to build a radiomics model for predicting axillary LNM in invasive ductal breast cancer, with an AUC of 0.890. Our results align with these studies, highlighting XGBoost’s effectiveness in radiomics-based LNM prediction.

Preceding investigations have elucidated the prospective efficacy of radiomics in prognosticating the survival outcomes of individuals with cervical cancer, boasting superior accuracy compared to traditional clinical parameters [40, 41]. Our study further explored the prognostic implications of MRI-derived radiomic characteristics. Kaplan-Meier analysis revealed that patients with higher radiomics scores exhibited significantly worse PFS, with a hazard ratio (HR) of 1.9 (95% CI: 1.2–3.0, *p* = 0.006). This suggests that radiomics-based risk stratification may offer valuable prognostic insights, aiding in personalized treatment planning. The ability to accurately predict disease progression using radiomics is crucial for clinical decision-making, as it can help tailor treatment strategies, such as determining the necessity of extended lymphadenectomy or adjuvant therapy for high-risk patients.

Despite the positive results of this research, it is also fundamental to consider the limitations of this study. This study was a retrospective, single-center study, which may introduce selection bias. External validation using multi-center datasets is needed to enhance the generalizability of our findings. Additionally, LNM was present in only 24.3% of cases, which could lead to class imbalance issues, potentially affecting model performance. Future studies should consider employing data augmentation or synthetic minority over-sampling techniques (SMOTE) to address this issue. Another limitation is the manual segmentation of the tumor ROI, which is time-consuming and subject to inter-observer variability. Automated segmentation methods, such as deep learning-based U-Net models, could improve reproducibility and reduce variability in feature extraction [42, 43]. Although our study demonstrated the superior performance of XGBoost, DL approaches, such as convolutional neural networks (CNNs), may further enhance predictive accuracy by automatically extracting high-level imaging features. Future research should explore hybrid models that integrate CNNs with traditional ML techniques to optimize predictive performance.

## CONCLUSION

In conclusion, ML is a step towards precision medicine in the field of gynecologic oncology. The preoperative assessment of the LNs is important for accurately staging patients with cervical cancer and for selecting the most suitable treatment. Our findings suggest that MRI-based radiomics analysis, particularly using the XGBoost model, holds promise for the preoperative prediction of LNM and prognosis in cervical cancer patients. This approach could be integrated into clinical workflows to aid radiologists and oncologists in more accurately assessing LNM risk, thereby facilitating more informed surgical and therapeutic decisions.

## Figures and Tables

**Fig. (1):**
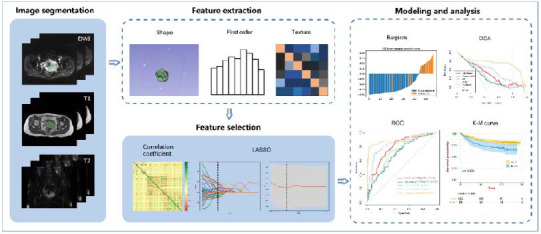
Artificial intelligence workflow and study flowchart.

**Fig. (4):**
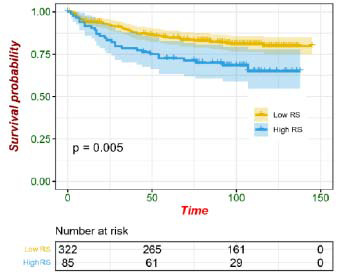
Kaplan-Meier curves of survival probability between the low and high radiomics score (RS) in all patients.

**Table 1:** Clinical characteristics of patients in training and validation cohorts.

**Characteristics**	**Training (n=284)**	**Validation(n=123)**	***P*-value**
Age, mean ± sd	47.16±8.304	47.25±7.885	0.197
Macroscopic type, n (%)	-	-	0.343
NT	148(52.1%)	72(58.5%)	-
CT	134(47.2%)	51(41.5%)	-
PTC	2(0.7%)	0(0.0%)	-
Tumor size (cm), n (%)	-	-	0.235
>4	159(56.0%)	61(49.6%)	-
≤4	125(44.0%)	62(50.4%)	-
Postoperative pathological, n (%)	-	-	0.393
SCC	265(93.3%)	110(89.4%)	-
AC	17(6.0%)	12(9.8%)	-
SAC	2(0.7%)	1(0.8%)	-
Differentiation degree, n (%)	-	-	0.588
Low grade	64(22.5%)	25(20.3%)	-
Middle grade	215(75.7%)	94(76.4%)	-
High grade	5(1.8%)	4(3.3%)	-
Depth of tumor invasion, n (%)	-	-	0.619
No	22(7.7%)	8(6.5%)	-
Near total	63(22.2%)	21(17.1%)	-
Superficial	93(32.7%)	45(36.6%)	-
Deep	106(37.3%)	49(39.8%)	-
Corpus invasion, n (%)	-	-	0.991
No	261(91.9%)	113(91.9%)	-
Yes	23(8.1%)	10(8.1%)	-
Parametrial invasion, n (%)	-	-	0.38
No	272(95.8%)	120(97.6%)	-
Yes	12(4.2%)	3(2.4%)	-
Vaginal invasion, n (%)	-	-	0.271
No	263(92.6%)	118(95.9%)	-
Yes	21(7.4%)	5(4.1%)	-
LNM, n (%)	-	-	0.786
No	216(76.1%)	92(74.8%)	-
Yes	68(23.9%)	31(25.2%)	-

**Abbreviations:** NT, Nodular Type; CT, Cauliflower-like Type; PTC, Postoperative Pathological Type Change; SAC, adenosquamous carcinoma; AC, adenocarcinoma; SCC, squamous cell carcinoma; LNM, lymph node metastasis.

**Table 2:** Radiomics signature selection results with descriptions.

**Feature**	**LASSO coefficient**
ceT1WI_original_glrlm_RunVariance	0.029388
ceT1WI _log-sigma-1-0-mm-3D_firstorder_90Percentile	-0.00053
ceT1WI _squareroot_firstorder_10Percentile	0.184889
T2WI_original_shape_Elongation	0.086653
T2WI _original_shape_Flatness	0.045143
T2WI _original_glcm_Imc1	0.032342
T2WI _log-sigma-1-0-mm-3D_firstorder_Kurtosis	0.207755
T2WI _log-sigma-1-0-mm-3D_glcm_ClusterShade	0.003092
T2WI _log-sigma-3-0-mm-3D_firstorder_Skewness	0.072891
T2WI _wavelet-LLH_firstorder_Maximum	-0.02597
T2WI _wavelet-HLL_firstorder_Minimum	0.266135
DWI_original_firstorder_90Percentile	-0.00246
DWI_log-sigma-1-0-mm-3D_firstorder_Skewness	0.150593
DWI_wavelet-LLH_firstorder_Kurtosis	0.108992
DWI_wavelet-HLL_firstorder_Skewness	-0.03973
DWI_wavelet-HHL_firstorder_Maximum	0.050664
DWI_exponential_glcm_Imc1	0.150122
DWI_squareroot_ngtdm_Coarseness	-0.01102

**Table 3:** Evaluation indicators of predictive performance of five models.

**Classifier**		**ACC**	**AUC**	**SEN**	**SPE**	**PPV**	**NPV**	**PRE**	**F1**
AdaBoost	Training	0.6620	0.7862	0.8824	0.5926	0.4054	0.9412	0.4054	0.5556
-	Validation	0.6829	0.7370	0.7419	0.6703	0.4259	0.8841	0.4259	0.5412
NB	Training	0.5563	0.7044	0.8529	0.4630	0.3333	0.9091	0.3333	0.4793
-	Validation	0.7236	0.7342	0.7419	0.7174	0.4694	0.8919	0.4694	0.5750
LR	Training	0.6901	0.7271	0.7206	0.6806	0.4153	0.8855	0.4153	0.5269
-	Validation	0.6667	0.7149	0.6774	0.6630	0.4038	0.8592	0.4038	0.5060
SVM	Training	0.8486	0.8386	0.7059	0.8935	0.6761	0.9061	0.6761	0.6906
-	Validation	0.7398	0.7907	0.8065	0.7174	0.4902	0.9167	0.4902	0.6098
XGBoost	Training	0.9296	0.9054	0.8088	0.9676	0.8871	0.9414	0.8871	0.8462
-	Validation	0.9268	0.8969	0.7419	0.9891	0.9583	0.9192	0.9583	0.8364

**Abbreviations:** AdaBoost, adaptive boosting; NB, Naïve Bayes; LR, Logistic Regression; SVM, support vector machine; XGBoost, extreme gradient boosting; ACC, accuracy; AUC, area under receiver operating characteristic curve; SEN, sensitivity; SPE, specificity; PPV, positive predictive value; NPV, negative predictive value; PRE, precision.

## Data Availability

The datasets generated during and analysed during the current study are available from the corresponding author [Q.X] on reasonable request.

## LIST OF ABBREVIATIONS

LN: Lymph Node
PD-L1: Programmed Death Ligand 1
US: Ultrasound
MRI: Magnetic Resonance Imaging
CT: Computed Tomography
IoT: Internet Of Things
AI: Artificial Intelligence
ML: Machine Learning
SVM: Support Vector Machine
T2WI: T2-Weighted Imaging
DWI: Diffusion-Weighted Imaging
GTV: Gross Tumor Volume
NIfTI: Neuroimaging Informatics Technology Initiative
NB: Naïve Bayes
LR: Logistic Regression
DCA: Decision Curve Analysis
ROC: Receiver Operating Characteristic Curve

## STANDARDS OF REPORTING

STROBE guidelines were followed.
