# Supplementary material for: Machine Learning based Radiomics from Multi-parametric Magnetic Resonance Imaging for Predicting Lymph Node Metastasis in Cervical Cancer
Source: Curr Med Imaging. 2025 Sep 18;21:e15734056376718. doi: 10.2174/0115734056376718250904221020 (PMC13096877; doi:10.2174/0115734056376718250904221020)
Supplement: Supplementary file 1 — Supplementary material is available on the publisher’s website along with the published article [file CMIM-21-E15734056376718_SD1.pdf]

1573-4056/25 *Send Orders for Reprints to [reprints@benthamscience.net](mailto:reprints@benthamscience.net)*



BENTHAM SCIENCE

CrossMark

# Current Medical Imaging

Content list available at: https://benthamscience.com/journals/cmir

## Supplementary Material

# Machine Learning based Radiomics from Multi-parametric Magnetic Resonance Imaging for Predicting Lymph Node Metastasis in Cervical Cancer

Jing Liu[1,#], Mingxuan Zhu[1,#], Li Li[1], Lele Zang[1], Lan Luo[1], Fei Zhu[1], Huiqi Zhang[1] and Qin Xu[1,*]

[1]*Departments of Gynecology, Clinical Oncology School of Fujian Medical University, Fujian Cancer Hospital (Fujian Branch of Fudan University Shanghai Cancer Center), Fuzhou 350014, Fujian, China*

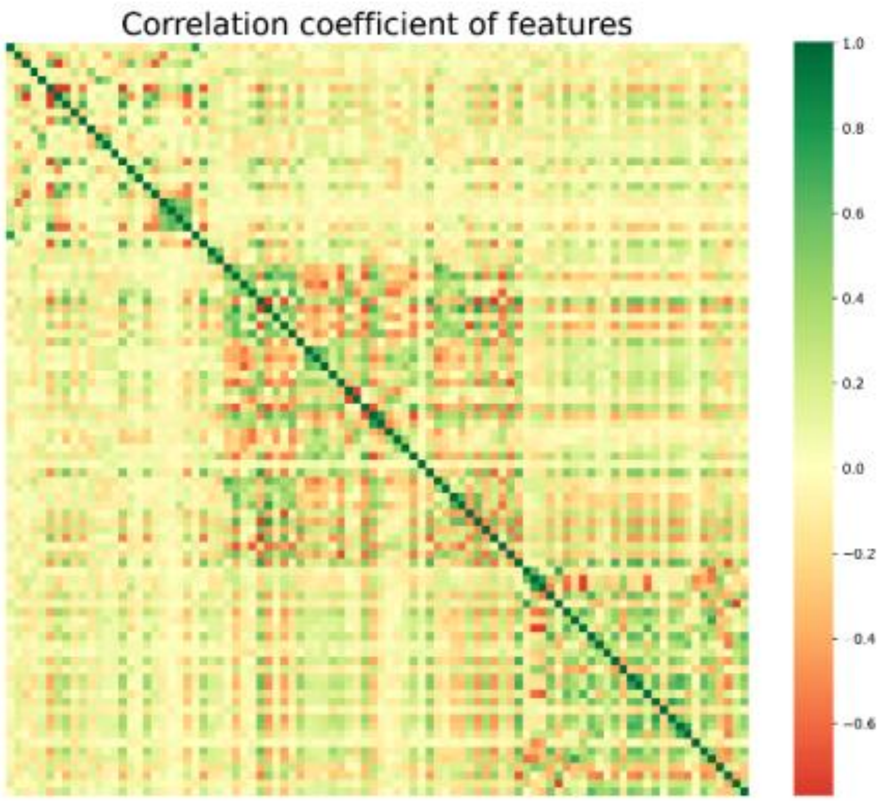


**Fig. (S1).** The correlation between MRI radiomic feature. Correlation analysis was used to estimate the strength of the correlations with Spearman p.

© 2025 The Author(s). Published by Bentham Science Publisher.

This is an open access article distributed under the terms of the Creative Commons Attribution 4.0 International Public License (CC-BY 4.0), a copy of which is available at: https://creativecommons.org/licenses/by/4.0/legalcode. This license permits unrestricted use, distribution, and reproduction in any medium, provided the original author and source are credited.

DOI: 10.2174/0115734056376718250904221020, 2025, *21*, e15734056376718
